# Supplementary material for: Comparative multiomics analysis of cell physiological state after culture in a basket bioreactor
Source: Sci Rep. 2022 Nov 23;12:20161. doi: 10.1038/s41598-022-24687-4 (PMC9686226; doi:10.1038/s41598-022-24687-4)
Supplement: Supplementary file 1 — Supplementary Information 1. [file 41598_2022_24687_MOESM1_ESM.zip › raw data/Transcriptomic raw data/6.Enrichment/1.GO/TE1vsTC1/DOWN/TE1vsTC1.down_GObar.pdf]

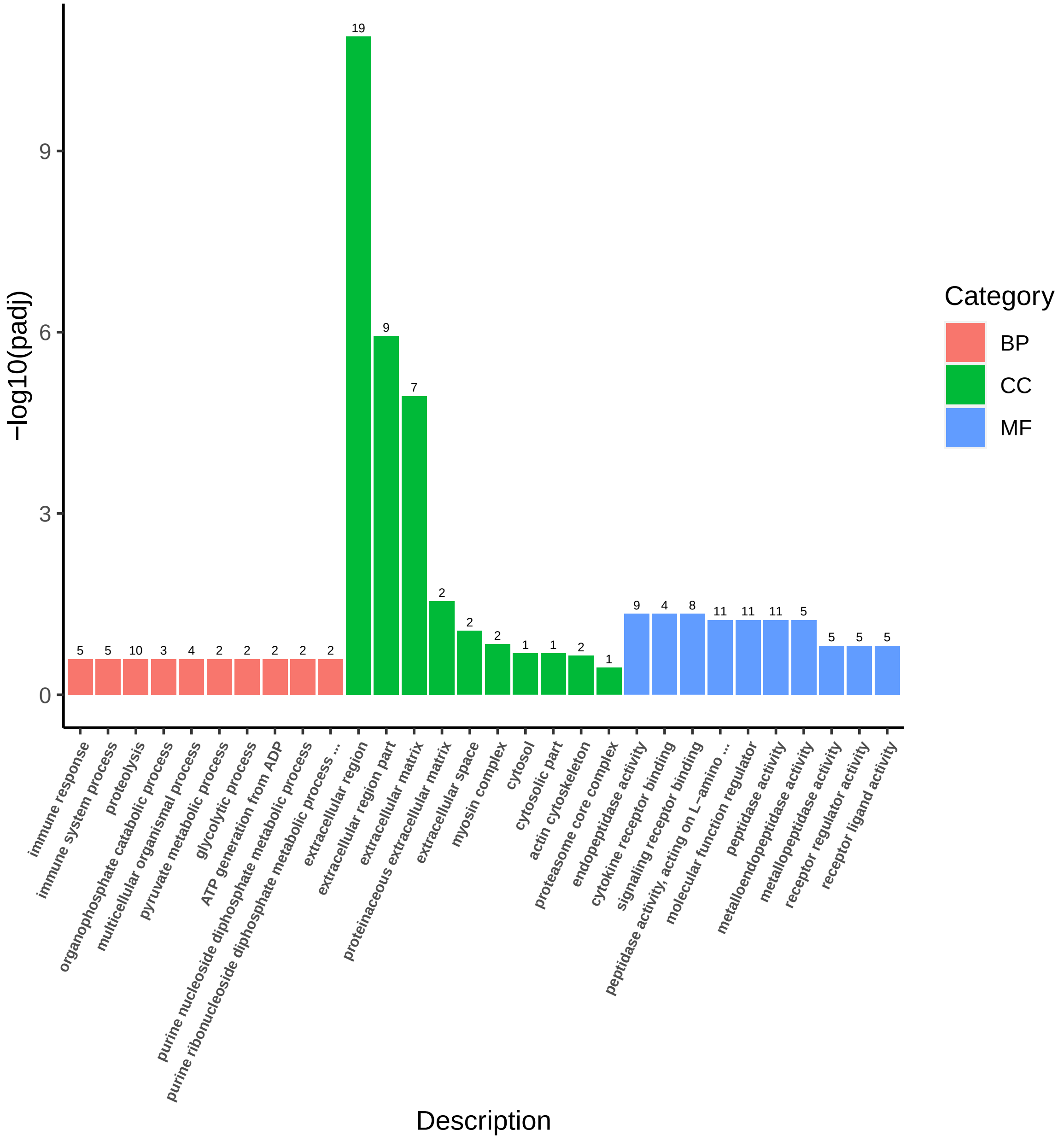

−log10(padj)
0
3
6
9
19
9
7
2
2
2
1
1
2
1
5
5
10
3
4
2
2
2
2
2
9
4
8
11
11
11
5
5
5
5
immune response
immune system process
proteolysis
organophosphate catabolic process
multicellular organismal process
pyruvate metabolic process
glycolytic process
ATP generation from ADP
purine nucleoside diphosphate metabolic process
purine ribonucleoside diphosphate metabolic process ...
extracellular region
extracellular region part
extracellular matrix
proteinaceous extracellular matrix
extracellular space
myosin complex
cytosol
cytosolic part
actin cytoskeleton
proteasome core complex
endopeptidase activity
cytokine receptor binding
signaling receptor binding
peptidase activity, acting on L−amino ...
molecular function regulator
peptidase activity
metalloendopeptidase activity
metallopeptidase activity
receptor regulator activity
receptor ligand activity
Description
Category
BP
CC
MF
